# An Asymptomatic Multiple Magnet Ingestion with Transmesenteric Entero-Enteric Fistula

**Published:** 2014-05-21

**Authors:** Federica Pederiva, Codrich Daniela, Maria-Grazia Scarpa, Edoardo Guida, Danica Dragovic, Stefano Martelossi

**Affiliations:** 1 Pediatric Surgery, Institute for Maternal and Child Health - IRCCS “Burlo Garofolo” – Trieste, Italy;; 2 Division of Pediatrics, Hospital of Monfalcone, Italy;; 3 Pediatrics, Institute for Maternal and Child Health - IRCCS “Burlo Garofolo” – Trieste, Italy;

**Keywords:** Intestinal perforation, Magnets, Enteric fistula

## Abstract

Ingestion of foreign bodies is a common presenting complaint in the pediatric emergency department. We present a case of a child in whom disc battery ingestion was suspected initially. The immobility of the foreign body on few days of conservative management raised the suspicion of two magnets. At operation, two magnets were found in the bowel causing a transmesenteric entero-enteric fistula.

## INTRODUCTION

Ingestion of foreign materials is frequent in children. For most of foreign bodies, the treatment is conservative, allowing safe passage of these objects through the intestinal tract. However, few foreign bodies require special considerations, such as button batteries that are potentially caustic and multiple magnets which attract each other while they are inside the gastrointestinal tract and could lead to subsequent pressure necrosis of the intestinal wall in between them, perforation, fistula formation and intestinal obstruction.[1-5] We report a case of un-witnessed ingestion of a foreign body by a child that turned out to be a diagnostic challenge.

## CASE REPORT

A 4-year-old boy was admitted to our hospital with vomiting of pieces of paper and no history of foreign body ingestion witnessed by the family. While the physical examination was unremarkable, the abdominal x-ray showed a disc-shaped foreign body (Fig. 1 A). The family did not recognize the object, but since they had moved to a new apartment, a lot of hypotheses were raised and it was finally thought to be a disc battery. The parents were not able to define the time of the ingestion. As the battery seemed beyond the stomach and there were no evidence of serious complications, expectant treatment was planned awaiting spontaneous passage. The child received polyethylene glycol colonoscopy preparation to expedite the progression of the foreign body through the intestine. After two days, he was still asymptomatic. The foreign body was not passed in the stool and its position on radiograph did not change. Moreover, on lateral x-ray abdomen a thin separating line was seen inside the foreign body (Fig. 1 B) that raised the suspicion of two magnets ingestion with a bowel wall lying in between them. An exploratory video-assisted laparoscopy was performed and an abnormal cluster of bowel loops was seen. To avoid the risk of perforation pulling the bowel loops which adhered tightly to each other, the small bowel was exteriorized through the umbilicus. At 70 cm distal to the ligament of Treitz, two ileal loops were noted adherent to the opposite sides of the mesentery of small bowel at the same point on their antimesenteric aspects. Further exploration revealed a transmesenteric entero-enteric fistula (Fig. 2). The fistula was isolated from the mesentery and resected and the antimesenteric borders of the two ileal loops were repaired. On opening the fistula on its longitudinal axis, two small magnets were found (Fig. 2). The patient’s parents recalled that the boy had played with the cell phone and the two magnets, which used to close the cover, were missing. The postoperative recovery was uneventful, and the patient discharged on 5th day of surgery.

**Figure:**
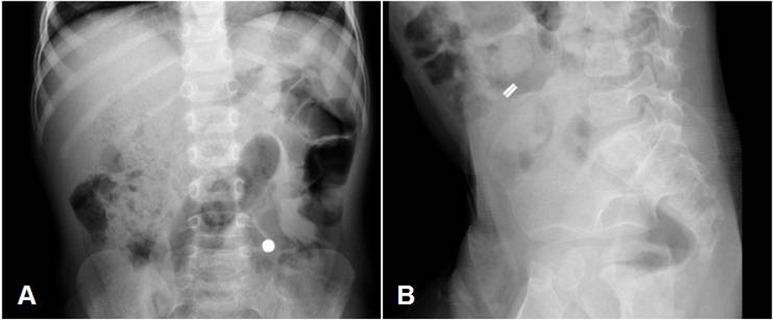
Figure 1: A.Abdominal X-ray showing a disc-shaped foreign body in the left lower quadrant. B.Lateral abdominal X-ray in which a separating line was noted inside the foreign body.

**Figure:**
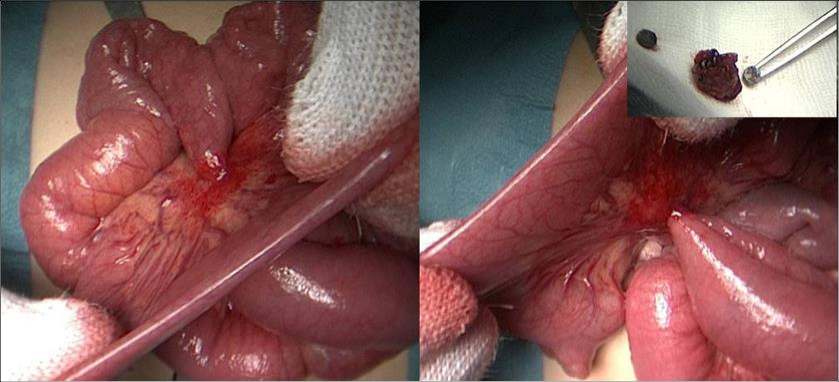
Figure 2: Intraoperative image showing the transmesenteric entero-enteric fistula and the magnets found inside the fistula.

## DISCUSSION

Most cases of foreign body ingestion occur in children between 6 month and 3 year of age. In about 80% of cases the objects pass the pyloric sphincter and the ileocecal valve and are naturally eliminated; 20% requires endoscopic removal and in 1% of the cases complications occur and demand surgical procedure.[2] Among ingested foreign materials, magnets require special concern because of the hazardous complications that may be produced. Even though a single swallowed magnet can be managed conservatively and generally passes spontaneously, things get complicated when two or more magnets are separated along their course in the gastrointestinal tract and may attract each other across bowel walls, holding the intestinal walls between them and causing pressure necrosis, with subsequent small bowel obstruction, volvulus, fistula formation, or perforation [1,4,5]. Our case was unique as the child remained asymptomatic throughout the preoperative period in spite the magnets produced bowel entrapment and a subsequent necrosis of two bowel lumens and complete wall of intervening mesentery to form a transmesenteric entero-enteric fistula. The child never complained abdominal pain or symptoms of intestinal obstruction; moreover, the physical examination was unremarkable. For these reasons the diagnosis was delayed. Once magnet ingestion is suspected, multiple radiologic views are recommended as it is possible for magnets to stick together, overlap on a single view, and be misdiagnosed as a single magnet or a different rod-like radiopaque foreign body, as happened in our case. A fixed position of the foreign body on follow-up x-rays should raise the suspicion of ingestion of more than one magnet and the possibility of an entrapped enteral wall between them even in asymptomatic patients.[3,5]

The real challenge for the physicians in cases of magnet ingestion is to find the right balance between the conservative management that is reasonable at the beginning suspecting a single magnet. Multiple magnets ingestion needs to close monitoring.

## Footnotes

**Source of Support:** Nil

**Conflict of Interest:** None declared

